# From fragmentation to coordination: strengthening One Health research to support H5N1 preparedness in Cambodia

**DOI:** 10.1016/j.ijregi.2026.100967

**Published:** 2026-07-31

**Authors:** Sao Puth, Luis Sagaon-Teyssier, Chanroth Chhoung, Chhavarath Dary, Dyna Khuon, Kennarey Seang, Vonthanak Saphonn

**Affiliations:** 1 The Global Health Research Center (GHRC), University of Health Sciences (UHS), 73 Preah Monivong Blvd, Sangkat Sras Chok, Khan Daun Penh, Phnom Penh, Cambodia; 2 Aix Marseille Univ, Inserm, IRD, SESSTIM, Sciences Economiques & Sociales de la Santé & Traitement de l’Information Médicale, ISSPAM, Marseille, France

**Keywords:** Avian influenza A (H5N1), One Health, Technical working group, Research integration, Cambodia

## Abstract

**Objectives:**

Highly pathogenic avian influenza A (H5N1) remains a major zoonotic threat, characterized by persistent transmission in Cambodia since its re-emergence in 2023. Despite strengthened surveillance and the establishment of the Inter-Ministerial Coordination Committee on One Health, limited integration of research across sectors constrains preparedness and response. This viewpoint examines how research supports the One Health system in Cambodia.

**Methods:**

This viewpoint draws on insights obtained from the first national multistakeholder workshop on H5N1, held in March 2026.

**Results:**

Fragmentation across epidemiological, clinical, behavioral, environmental, and genomic domains limits the generation of actionable evidence and delays its translation into policy.

**Conclusion:**

We propose the establishment of a multisectoral technical working group on H5N1 research embedded within the Inter-Ministerial Coordination Committee on One Health to align research priorities, strengthen data integration, and improve evidence-to-policy translation. This approach could enhance national preparedness while simultaneously positioning Cambodia as a model for coordinated One Health research in the Western Pacific region and beyond.

## Highlights

• Persistent H5N1 transmission in Cambodia reflects unresolved One Health gaps.
• Limited interoperability constrains multisectoral surveillance and research.
• Integrated epidemiological, clinical, genomic, and behavioral data are needed.
• A multisectoral H5N1 research working group is proposed in Cambodia.
• Coordinated research may improve preparedness for emerging zoonotic threats.

## Introduction

Highly pathogenic avian influenza A (H5N1) remains a significant zoonotic threat and a major concern for global health [1]. Since this disease was first detected in humans in 1997 in Hong Kong [2], H5N1 has spread across 24 countries between 2003 and July 2025, exhibiting a case fatality rate of nearly 50% in 2025 [3]. The link with human infections is explained principally by the exposure of domestic poultry to the virus, which is carried by migratory waterfowl [4,5]. The genetic diversification of the virus highlights its pandemic potential, as demonstrated by clade 2.3.4.4b, which affected more than 43 species of mammals, particularly through the recent unusual infections in cows in 2024 [1,4,[6], [7], [8]].

The Western Pacific region is characterized by recurrent spillovers associated with a high density of poultry, large groups of ducks, and close human-animal interactions [9,10], as well as human settlements that increase the degree to which people are exposed to the virus [11,12]. In Cambodia, the first case of H5N1 was recorded in 2003, followed by a period of relative epidemiological silence between 2014 and 2022 and the re-emergence of the virus in 2023, resulting in a cumulative case fatality rate of 59% [49/83 cases] as of 1 July 2025 [13,14]. Overall, children and individuals who live in rural areas were affected most frequently, and most cases were associated with close contact with sick or dead poultry [14,15]. The epidemic in Cambodia was characterized by an endemic lineage identified in 2010 (clade 1.1A) and subsequent cycles of clades (2.3.2.1c in the post-2014 period and 2.3.4.4b since 2016), thereby suggesting both specific transmission patterns and regional adaptation of the virus [14]. Live bird markets, particularly poultry stock management and flows, have been identified as crucial drivers of H5N1 epidemic dynamics in the country, although unsafe poultry handling practices, a lack of awareness, and socioeconomic and behavioral factors have also played important roles in the spread and perpetuation of the virus [[16], [17], [18]].

The persistent circulation of H5N1 in Cambodia and the socioecological specificities underlying its epidemic dynamics have given rise to significant improvements in terms of surveillance. In 2023, Cambodia’s national strategy for H5N1 detection, which relies on event-based surveillance (i.e. testing contacts of dead poultry, clinical suspicion, and active case-finding among close contacts), was strengthened through the addition of sentinel surveillance for severe acute respiratory infections [19,20]. However, several challenges remain with regard to the timely detection and management of H5N1 cases. While delays in health care seeking and diagnosis contribute to poor clinical outcomes at the individual level [19,21], gaps in access to screening and diagnostic tools, variable health care provider awareness, and other structural limitations within the health system hinder the early identification of cases [14,16,19]. Furthermore, the predominance of severe, hospitalized cases among confirmed infections suggests that mild or asymptomatic infections remain underdetected, which may bias estimates of disease severity [19]. Efforts to address these challenges require narrowing knowledge gaps that could reveal transmission patterns specific to Cambodia while simultaneously contributing to responses to H5N1 both in the region and globally. This process involves understanding additional transmission patterns, such as oral ingestion and conjunctival or intranasal contamination [22]; improved monitoring of potential environmental vectors; and the roles played by genetic and immunological factors throughout the clinical spectrum of H5N1 [15,[23], [24], [25]]. More broadly, understanding the dynamics of H5N1 animal-to-human and potential human-to-human infections requires the adoption of a comprehensive approach that can offer a better understanding of the interactions among virological, environmental, clinical, socioecological, socioeconomic, behavioral, and cultural factors.

In an effort to address persistent gaps in the early detection and case management of zoonotic diseases, including H5N1, an Inter-Ministerial Coordination Committee on One Health (IMCC-OH) was established in 2023. In comparison with other countries in the region, the IMCC-OH in Cambodia is notable because of its formalized, government-endorsed structure, which provides multiple ministries with a single coordination platform, thus facilitating consistent collaboration and joint decision-making. Its multidisciplinary nature improves information sharing and strengthens outbreak response capacity through the integration of human, animal, and environmental health expertise. However, grounding these efforts in coordinated interdisciplinary research is essential for informing the IMCC-OH, supporting evidence-based priorities, improving response strategies, and guiding public policy.

In this context, to explore how research could support the activities of the IMCC-OH, a consultative workshop on H5N1 research was organized for the first time in Cambodia. This workshop united the technical departments of the Ministries of Health, Agriculture, Forestry and Fisheries, and Environment, alongside national and international research teams, academics, representatives of national hospitals, nongovernmental organizations, civil society, and international funding agencies. The workshop aimed to facilitate knowledge sharing concerning ongoing programmatic and research activities related to H5N1 in Cambodia, to identify gaps in surveillance and response, to define priority research questions within the One Health approach, and to develop an actionable plan to align research efforts more closely with national priorities and strengthen the activities of IMCC-OH. This viewpoint argues that, despite the establishment of a formal One Health coordination mechanism in Cambodia, research activities remain fragmented both across and within sectors, thus limiting their impact on preparedness and responses to H5N1. We draw on a structured thematic synthesis of the presentations and multidisciplinary discussions from the first national multistakeholder workshop on H5N1 in Cambodia to highlight critical gaps in research integration and alignment with policy.

We propose that the establishment of a coordinated, multisectoral research technical working group could strengthen data sharing, improve the translation of evidence into policy to inform the IMCC-OH, and enhance the alignment of national priorities with international funding agendas, thereby increasing access to funding and strengthening the potential impact of Cambodia’s efforts at both the regional and global levels.

## Research integration within the One Health mechanism and national ownership in Cambodia

The experience of Cambodia should be interpreted within the broader landscape of One Health governance. A structured comparative analysis of national One Health mechanisms across 49 countries was conducted using predefined indicators of research integration and national ownership (Supplementary Material 1). The comparative framework was developed to provide a structured descriptive assessment of governance arrangements rather than to generate quantitative rankings or infer a causal relationship between governance characteristics. The analysis revealed substantial heterogeneity in the extent to which research is formally integrated into national One Health coordination mechanisms. Countries with highly institutionalized One Health systems, such as Australia, China, Japan, and Singapore, generally demonstrated greater formal integration of research within their governance structures. In contrast, in several countries where One Health mechanisms remain only partially institutionalized, research activities continue to rely primarily on project-based or externally supported initiatives, with fewer formal mechanisms linking research, surveillance, and policy.

According to the structured comparative framework (Supplementary Material 1), Cambodia shows moderate national ownership and mild research integration within its One Health governance mechanism. While the establishment of the IMCC-OH reflects increasing institutional commitment, the integration of research within the One Health mechanism remains only partially institutionalized. Research actors participate intermittently, cross-sectoral data sharing remains limited, and formal mechanisms linking research, evidence synthesis, and policy development are still evolving. As a result, research and surveillance activities continue to operate largely in parallel rather than as components of a fully integrated One Health system. This pattern is consistent with those observed in other countries in the Western Pacific region, including Laos, Malaysia, and the Philippines, where coordination structures for research exist but remain only partially institutionalized within the One Health mechanism. Surveillance platforms across the human, animal, and environmental sectors are not yet interoperable, and standardized procedures for data sharing remain limited. Consequently, although substantial data and research expertise exist, institutional coordination has not yet translated into integrated research and evidence use.

## The gap: the fragmentation of research in practice

Cambodia has developed a multilayered surveillance system that combines indicator-based (influenza-like illness, severe acute respiratory infection, live bird markets, and genomic surveillance of acute febrile illness) and event-based (community alerts, hotline reporting, rapid notification, and event monitoring) information with the support of national coordination facilitated by the Department of Communicable Disease Control. However, despite these advances, research activities remain insufficiently integrated both within and across sectors. Surveillance systems across human, animal, and environmental health continue to operate in parallel; the integration of data across sectors remains limited, and few formal mechanisms link data generation to coordinated research agendas or syntheses of evidence. The engagement of research actors remains intermittent, thereby further constraining integration. This fragmentation extends to the connection between research and clinical practice. H5N1 infection is associated with severe disease and high mortality, but gaps in research integration limit the systematic analysis of clinical outcomes, treatment effectiveness, and the drivers of delayed care. Variability in clinical practices across facilities further reflects the limited use of shared evidence and weak connections among surveillance, research, and clinical decision-making processes.

Fragmentation also affects the broader One Health research landscape. H5N1 transmission in Cambodia is shaped by complex interactions among wildlife, livestock, human populations, and environmental conditions, including migratory bird pathways and climate-sensitive ecosystems. However, these dimensions remain insufficiently integrated into surveillance and research activities, which continue to operate largely within sectoral boundaries. Environmental and ecological data have not yet been routinely incorporated into integrated analyses, and socioeconomic and behavioral factors among communities and health care providers remain insufficiently studied despite their relevance to transmission dynamics and clinical outcomes.

## Why does the fragmentation of research matter?

The workshop generated a broad range of multidisciplinary presentations and discussions on H5N1 preparedness in Cambodia. Following the workshop, detailed notes from the presentations and discussions were reviewed by the author group and subjected to a structured thematic synthesis. Through an iterative analytical process, recurrent themes, areas of convergence across sectors, and priority research gaps were identified and organized into six cross-cutting domains. These domains formed the basis of both the workshop’s scientific report and the analytical framework presented in Table 1. Rather than representing the findings of a formal qualitative research study, Table 1 provides an expert-informed synthesis of the principal research gaps and priorities emerging from this national multisectoral consultation.

**Table 1:** Structured thematic synthesis of the principal research gaps and priorities emerging from the national multisectoral H5N1 workshop.

Thematic area	Key insight	Implications for research and policy
Epidemiological context	Cambodia remains a hotspot for H5N1, and zoonotic transmission remains ongoing following its recent resurgence in 2023, after a period of epidemiological silence (2014-2022).	▪ Establish coordinated national research priorities to obtain a better understanding of temporal dynamics (including periods of apparent absence and resurgence); ▪ Strengthen the integration of surveillance data across sectors to support continuous monitoring and early warning; ▪ Align research agendas with national preparedness strategies and international priorities.
Transmission dynamics and behaviors	Transmission is primarily zoonotic and driven by poultry exposure, live bird markets, and sociocultural and behavioral practices.	▪ Integrate socioeconomic and behavioral research into national research agendas to obtain a better understanding of exposure, risk perception, care-seeking behaviors, and outbreak reporting; ▪ Link agricultural, public health, and community-level data to inform the development of targeted prevention strategies; ▪ Strengthen risk communication and community engagement on the basis of evidence generated across sectors.
Viral evolution and risk	Ongoing viral reassortment and adaptation increase uncertainty and potential emergence risk, including possible changes in host range and transmissibility.	▪ Expand genomic surveillance and integrate it with epidemiological and field data; ▪ Strengthen interdisciplinary research on viral evolution, the phylogeography of avian influenza, reassortment, and cross-species transmission; ▪ Develop coordinated risk assessment frameworks that can link research findings to policy decision-making.
Clinical management and outcomes	High case fatality is associated with delayed detection, late presentation, and variability in clinical practices across facilities.	▪ Conduct coordinated clinical research to evaluate treatment effectiveness, the timing of antiviral use, and drivers of mortality; ▪ Standardize and strengthen adherence to clinical management protocols across facilities; ▪ Strengthen early detection, referral systems, and integration between surveillance and clinical care.
Surveillance and research integration	Surveillance systems operate in parallel across sectors, but this situation is characterized by limited integration of data, weak interoperability, and insufficient connections to research and policy processes.	▪ Develop interoperable surveillance systems that can facilitate cross-sectoral data sharing and joint analysis; ▪ Establish formal mechanisms that can link surveillance outputs to coordinated research agendas and evidence synthesis; ▪ Strengthen institutional platforms (e.g. TWGR-H5N1) to support coordination, data integration, collaboration, and translation into policy.
One Health and environmental factors	Environmental, wildlife, and ecological drivers of transmission are not fully integrated into surveillance, research, and response frameworks.	▪ Promote interdisciplinary One Health research that can integrate environmental, animal, and human health data; ▪ Incorporate spatial information, climatic, and ecological factors into surveillance and predictive modeling; ▪ Strengthen collaboration among environmental, veterinary, and public health sectors within coordinated research platforms.

Abbreviation: TWGR-H5N1, Technical Working Group on H5N1 Research.

The synthesis illustrates how the fragmentation of research across sectors constrains the effectiveness of preparedness and response. Key challenges identified during the workshop were organized into six domains: (i) epidemiological context; (ii) transmission dynamics and behaviors; (iii) viral evolution and risk; (iv) clinical management and outcomes; (v) surveillance and research integration; and (vi) One Health-related and environmental factors (Table 1). The gaps identified in research integration limit the ability to obtain a comprehensive and timely understanding of the disease’s epidemiological dynamics. In particular, the resurgence of cases following a period of apparent epidemiological silence highlights the need for coordinated research to characterize transmission patterns more accurately and detect early signals of re-emergence.

Fragmentation also limits our understanding of key drivers of transmission. While zoonotic exposure remains the primary transmission pathway, the insufficient integration of socioeconomic and behavioral research limits our ability to identify and address risky practices. The absence of coordinated, interdisciplinary research that links agricultural, environmental, and public health data remains a challenge, and prevention and care strategies remain only partially informed and are thus less effective. Moreover, the lack of integration between research and clinical practice affects patient outcomes. High case fatality rates are closely linked to delayed detection and variability in clinical management, but the absence of coordinated clinical research and data sharing limits the systematic evaluation of treatment effectiveness and the drivers of mortality. This limitation reduces our ability to standardize care and optimize early diagnosis and referral pathways across the health system. Fragmentation further undermines our ability to anticipate and respond to evolving risks. Ongoing viral evolution, including reassortment and adaptation, requires the integration of genomic, epidemiological, and field-based research to support real-time risk assessment. However, the disconnection among these components reduces opportunities for early warning and proactive response. Finally, the limited integration of environmental and ecological data into research and surveillance systems constrains the application of a full One Health approach. Environmental drivers, including wildlife dynamics and climate-sensitive factors, play a critical role in shaping transmission patterns, but they have not been systematically incorporated into predictive analyses or decision-making frameworks. As a result, overall responses remain reactive rather than anticipatory.

The challenges thus identified highlight the fact that the fragmentation of research is not only a technical issue but also a systemic constraint on preparedness. This problem limits our ability to generate actionable evidence, delays the translation of such evidence into policy, and reduces the effectiveness of coordinated responses across sectors. Efforts to address this fragmentation are therefore essential to the goal of strengthening both national and regional capacity to prevent and respond to H5N1, as well as to other emerging zoonotic threats.

## A way forward: coordinated One Health research

The task of addressing the fragmentation of research and evidence identified across H5N1 preparedness in Cambodia requires a more coordinated approach to research within the existing One Health system. The proposed Technical Working Group on H5N1 Research (TWGR-H5N1) is not intended to duplicate the multisectoral coordination role of the IMCC-OH. Rather, it is designed to address a complementary function that remains insufficiently institutionalized: the coordination of research and the translation of scientific evidence into policy. While the IMCC-OH provides the governance framework for multisectoral preparedness and response, research activities continue to be conducted largely independently across institutions, resulting in fragmented research agendas, limited data integration, and few formal mechanisms for evidence synthesis or knowledge translation. The proposed TWGR-H5N1 would therefore serve as a dedicated scientific coordination platform linking researchers, surveillance systems, and decision-makers. By coordinating research priorities, promoting multidisciplinary collaborations, facilitating evidence synthesis, and translating research findings into policy-relevant recommendations, the TWGR-H5N1 would strengthen the science-policy interface while supporting, rather than duplicating, the mandate of the IMCC-OH.

**Figure 1:**
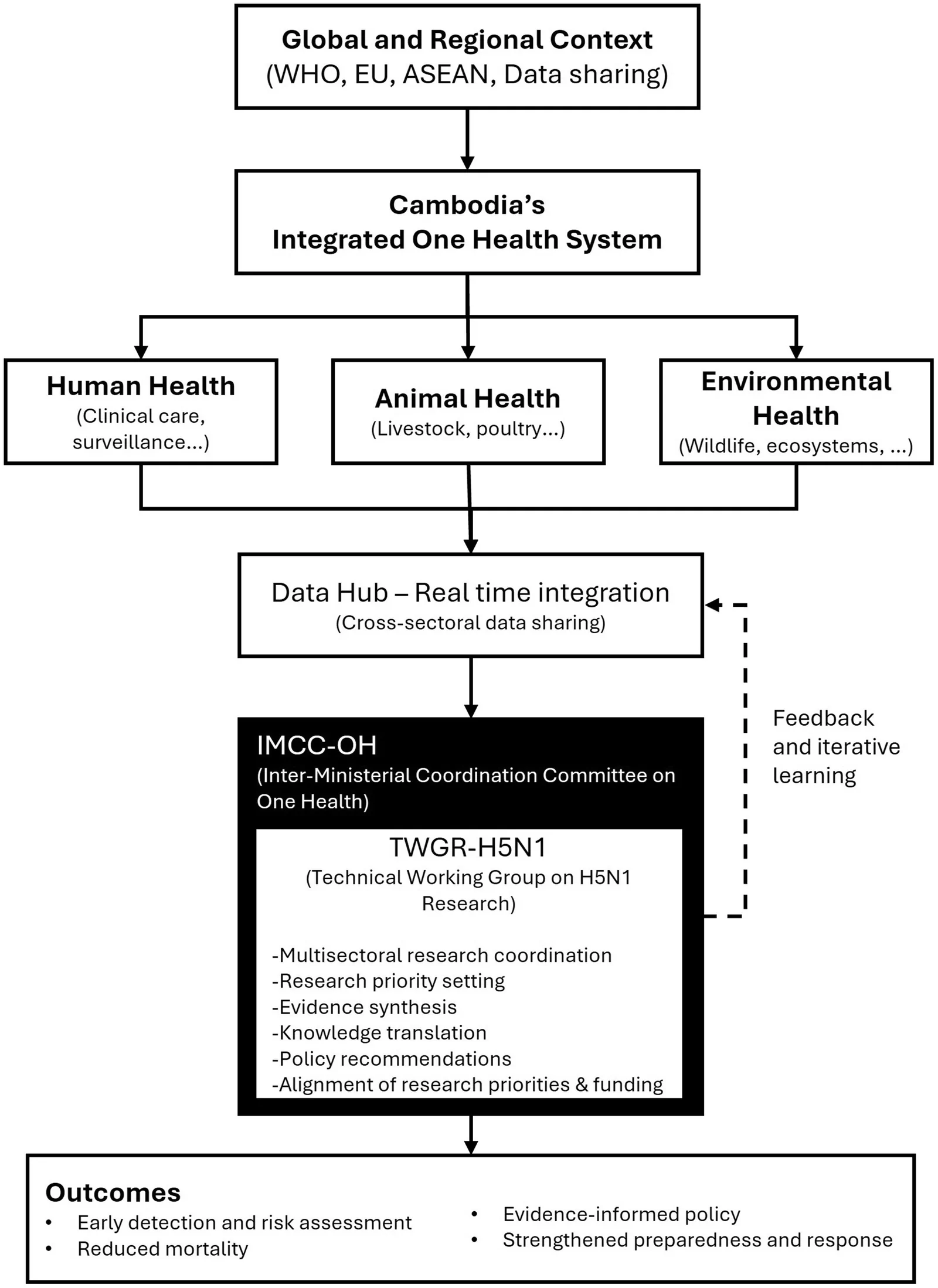
Conceptual One Health framework for H5N1 preparedness in Cambodia: place of the TWGR-H5N1.

Abbreviations: ASEAN, Association of Southeast Asian Nations; EU, European Union; IMCC-OH, Inter-Ministerial Coordination Committee on One Health; TWGR-H5N1, Technical Working Group on H5N1 Research; WHO, World Health Organization.

Despite the growing institutional commitment to multisectoral coordination through the establishment of the IMCC-OH, the integration of research within this framework remains limited. As highlighted through the workshop discussions, the absence of a structured mechanism to align and coordinate research activities constrains the ability to generate and use evidence effectively for preparedness and response. Figure 1 illustrates how a multisectoral TWGR-H5N1 could integrate data and research across sectors closely aligned with the IMCC-OH.

The establishment of a multisectoral TWGR-H5N1, as proposed during the workshop, could provide a dedicated scientific coordination platform operating at the interface between research and policy. Such a platform could support the identification and alignment of priority research areas based on the key gaps highlighted in epidemiological trends, transmission dynamics and behaviors, viral evolution and risk, clinical management, surveillance systems, and One Health interfaces. Such a TWGR-H5N1 could facilitate more consistent engagement by research actors and reduce the fragmentation of research efforts by uniting actors from the human, animal, and environmental health sectors, as well as research institutions and technical partners. A coordinated research platform could also help improve the integration of data and evidence generation across sectors. The discussions held at the workshop also highlighted persistent challenges in the context of linking surveillance data, research activities, and decision-making processes. Strengthening coordination through this TWGR-H5N1 could, therefore, support the more systematic use of available data, facilitate joint analyses across sectors, and improve our overall understanding of H5N1 risk and transmission in Cambodia. In addition, the regular engagement across sectors associated with the proposed TWGR-H5N1 could strengthen the connection between research and policy processes by providing feedback in both directions and subsequently contributing to the improved uptake of research evidence by policy-level decision-makers. The limited integration of research findings into decision-making within the IMCC-OH was identified as a key gap in this regard. A dedicated technical working group for research could support the synthesis of research outputs and their translation into policy-relevant information, thereby contributing to more evidence-informed preparedness and response strategies. Finally, the coordination of research activities at the national level could support better alignment with international funding priorities. As highlighted during the workshop, aligning national research needs with the priorities of international partners represents an opportunity to mobilize resources while simultaneously ensuring that externally supported activities contribute to national objectives. Efforts to strengthen coordination could, therefore, help reduce the fragmentation associated with parallel initiatives and support more coherent and sustainable research efforts.

The challenges identified in this viewpoint are not unique to Cambodia and reflect broader difficulties in integrating research within multisectoral One Health governance. Rather than creating an additional governance mechanism, the proposed TWGR-H5N1 is intended to fill a specific institutional gap by providing a dedicated platform for research coordination, evidence synthesis, and the translation of scientific findings into policy recommendations. By strengthening the science-policy interface, the TWGR-H5N1 would complement the existing mandate of the IMCC-OH and support more coordinated, evidence-informed preparedness and response. Although developed in the Cambodian context, this model may also provide useful lessons for other countries seeking to strengthen the integration of research within existing One Health coordination mechanisms.

The proposed TWGR-H5N1 would operate under the authority of the IMCC-OH. The Global Health Research Center (GHRC) at the University of Health Sciences would provide the technical secretariat, supporting meeting organization, communication, documentation, and administrative coordination, but would not have governance or decision-making authority. Once established, the Chair and membership of the TWGR-H5N1 would be determined by consensus among its members, ensuring multisectoral representation from government ministries, universities, research institutions, public health agencies, and technical partners. This governance arrangement is intended to ensure scientific independence, transparency, and accountability while strengthening the integration of research within the existing national One Health governance system.

## Conclusion

The resurgence of H5N1 in Cambodia since 2023 highlights the continued risk posed by zoonotic diseases at the human-animal-environment interface. Despite the progress that has been made in multisectoral coordination through the IMCC-OH, the limited integration of research across sectors remains a critical constraint to effective preparedness and response. The persistence of zoonotic transmission and ongoing viral evolution further emphasize the need for coordinated, multidisciplinary, and cross-sectoral research to anticipate and mitigate emerging risks more effectively. The establishment of a TWGR-H5N1 offers a practical mechanism that can help align research priorities, strengthen data integration across sectors, and support the translation of evidence into policy and practice. Such a TWGR-H5N1 could enhance the effectiveness and resilience of preparedness systems by improving coordination of research and subsequently increasing opportunities to access funding, as well as by reinforcing the links between research and decision-making within the IMCC-OH.

The central role of Cambodia in the current H5N1 epidemiological landscape also offers an opportunity to inform regional and global responses. The institutionalization of a coordinated research mechanism could represent a first step toward the goal of strengthening the science-policy connection within the One Health system. While increased research integration is closely linked to national ownership, it does not necessarily require highly formalized structures; sustained coordination and alignment of actors and priorities are key to the link between research, policy, and practice. If successfully implemented and evaluated, the proposed TWGR-H5N1 may provide useful lessons for other countries seeking to strengthen the integration of research within existing One Health governance mechanisms. Its broader applicability should be assessed through future implementation and evaluation in different institutional contexts.

## Positionality statement

Several authors are affiliated with the Global Health Research Center at the University of Health Sciences, which co-organized the national H5N1 workshop that informed this viewpoint. The perspectives presented in this article reflect the authors' academic interpretation of the workshop discussions and the broader literature on One Health governance. The proposed TWGR-H5N1 is intended to operate under the governance of the IMCC-OH. Within this model, the GHRC will provide technical secretariat support but will not hold governance or decision-making authority, which remains vested in the IMCC-OH and the TWGR-H5N1 through a transparent, multisectoral governance process.

## CRediT authorship contribution statement

**Sao Puth:** Writing – review & editing, Writing – original draft, Visualization, Investigation, Formal analysis. **Luis Sagaon-Teyssier:** Writing – review & editing, Writing – original draft, Visualization, Methodology, Investigation, Formal analysis, Conceptualization. **Chanroth Chhoung:** Writing – review & editing, Formal analysis. **Chhavarath Dary:** Writing – review & editing, Formal analysis. **Dyna Khuon:** Writing – review & editing, Formal analysis. **Kennarey Seang:** Writing – review & editing, Validation, Supervision, Methodology, Investigation, Conceptualization. **Vonthanak Saphonn:** Writing – review & editing, Validation, Supervision, Methodology, Investigation, Conceptualization.

## Declaration of competing interest

The authors have no competing interests to declare.

## Appendix Supplementary materials

Image, application 1

## Footnotes

Supplementary material associated with this article can be found, in the online version, at doi:10.1016/j.ijregi.2026.100967.
