# Supplementary material for: From fragmentation to coordination: strengthening One Health research to support H5N1 preparedness in Cambodia
Source: IJID Reg. 2026 Jul 31;20:100967. doi: 10.1016/j.ijregi.2026.100967 (PMC13562410; doi:10.1016/j.ijregi.2026.100967)
Supplement: Supplementary file 1 [file mmc1.docx]

**Supplementary material 1. Scoring and classification methodology**

We used a qualitative comparative approach involving the use of structured indicators to facilitate systematic comparisons across countries. This approach does not constitute a systematic review; rather, it represents a rapid review conducted on the basis of a structured, iterative search, including triangulation with publicly available government documents, internal reports, peer-reviewed and the grey literature. Searches were conducted on the basis of combinations of key words including “One Health”, “multisectoral coordination”, “interministerial committee”, “zoonotic disease”, “governance” and “antimicrobial resistance”, in combination with country names. These keywords were used to explore multiple sources including general search engines (e.g., Google), international organization websites, national government portals and academic databases (e.g., PubMed). The search of countries was not systematic but rather relied on a list constructed by reference to the World Health Organization (WHO), the Food and Agriculture Organization (FAO), and the World Organization for Animal Health (WOAH), as well as the One Health Commission and FAOLEX repositories, as well as peer-reviewed articles [1–3]. Official national strategies and institutional descriptions were prioritized. The absence of a country from this set of information does not imply the absence of One Health activities but rather reflects limitations in documentation or the absence of identifiable structures.

**Research integration** was assessed as a proxy for the strength of the science-policy interface within national One Health systems. Indicators were constructed on the basis of official frameworks and the evidence-informed policy literature, which have emphasized multisectoral coordination, integrated surveillance, and knowledge translation as core system functions [4–8]. Five dimensions were assessed:

i) Institutional integration of research actors: membership of researchers in coordination bodies or formal advisory roles.
ii) Use of evidence in policy processes: use of data, studies, and risk assessments, as well as formal evidence-review processes.
iii) Integration of surveillance systems: joint surveillance platforms and data-sharing mechanisms.
iv) Knowledge production: publications, surveillance outputs, and research programmes.
v) Policy-research feedback mechanisms: advisory scientific committees and formal consultation processes.

Each dimension was scored as 0 (no documented evidence), 1 (ad hoc use of research), or 2 (formalized or systematic integration). Composite scores ranged from 0 to 10, thereby indicating weak (2-4), mild (5-7) or strong (8-10) research integration with the One Health system.

**Examples of scores for the research integration composite indicator**

| Country | Institutional integration | Evidence use | Surveillance integration | Knowledge production | Policy-research feedback | Total score | Group |
| --- | --- | --- | --- | --- | --- | --- | --- |
| Cambodia | 1 | 1 | 1 | 1 | 1 | 5 | Mild |
| France | 2 | 2 | 2 | 2 | 2 | 10 | Strong |
| Sierra Leone | 1 | 1 | 1 | 0 | 0 | 3 | Weak |

National ownership was assessed as a four-level category that reflected domestic leadership and institutionalization on the basis of global health governance and aid-effectiveness frameworks [9–11]. Three indicators were considered:

i) Government leadership: leadership of coordination mechanisms, involvement of key ministries, and decision-making authority.
ii) Level of institutionalization: formal committees or units, legal or administrative mandates, and permanent secretariats.
iii) External technical or financial independence: donor funding and externally driven programmes.

Each indicator was scored as 0 (absent/weak), 1 (partial/moderate), or 2 (strong). Composite score ranged from 0 to 6, thereby indicating limited ownership (0-2, externally driven), emerging (3-4, government involvement with high external dependence), moderate (5, government-led but partially institutionalized), or strong (6, fully institutionalized and domestically led).

**Examples of scores for the national ownership composite indicator**

| Country | Government leadership | Institutionalization | External independence | Total | Classification |
| --- | --- | --- | --- | --- | --- |
| Cambodia | 2 | 1 | 1 | 4 | Moderate |
| France | 2 | 2 | 2 | 6 | Strong |
| Sierra Leone | 1 | 0 | 0 | 1 | Limited |

For both research integration and national ownership indicators, data were extracted manually and cross-checked across sources. In cases in which evidence was limited or inconsistent, a conservative scoring approach was used. The resulting classifications should be interpreted as structured comparative proxies rather than direct measures of system performance.

Available documents with identifiable One Health mechanisms were identified for 49 countries, including countries 13 in the WHO Western Pacific region (26.5%), 18 countries in the African region (36.7%), 8 countries in the Southeast Asia Region (16.3%), 4 countries in the European region (8.2%), 4 countries in the Americas region (8.2%), and countries 2 in the Eastern Mediterranean region (4.1%).

The purpose of this comparative framework was to provide a structured descriptive comparison of national One Health governance mechanisms rather than to generate quantitative rankings or infer causal relationships between governance characteristics.

**References**

[1]	Lâm S, Dang-Xuan S, Unger F, Meeyam T, Pham-Duc P, Wacharapluesadee S, et al. Operationalizing regional One Health initiatives in Southeast Asia: Ways forward. *One Health*;2025;20:101034. doi: 10.1016/j.onehlt.2025.101034.

[2]	Agbo S, Gbaguidi L, Biliyar C, Sylla S, Fahnbulleh M, Dogba J, et al. Establishing National Multisectoral Coordination and collaboration mechanisms to prevent, detect, and respond to public health threats in Guinea, Liberia, and Sierra Leone 2016–2018. *One Health Outlook*;2019;1:4. doi: 10.1186/s42522-019-0004-z.

[3]	Zhang Q, Liu J, Han L, Li X, Zhang C, Guo Z, et al. How far has the globe gone in achieving One Health? Current evidence and policy implications based on global One Health index. *Science in One Health*;2024;3:100064. doi: 10.1016/j.soh.2024.100064.

[4]	One health Joint Plan of Action (2022-2026). Working together for thehealth of humans, animals, plants and the environment. Rome: FAO; UNEP; WHO; World Organisation for Animal Health (WOAH) (founded as OIE); 2022. doi: 10.4060/cc2289en.

[5]	Taking Multisectoral, One Health Approach: A tripartite Guide to Addressing Zoonotic Diseases in Countries. Geneva: World Health Organization (WHO), Food and Agriculture Organization of the United Nations (FAO) and World Organisation for Animal Health (OIE),; 2019. Available from: https://iris.who.int/server/api/core/bitstreams/00a4190f-ff85-4959-b020-db11bca0565b/content. Accessed 2026 Apr. 24.

[6]	Scientific Advice Mechanism to the European Union. One Health governance in the European Union: Evidence review report. SAPEA; 2024. doi: 10.5281/ZENODO.14055991.

[7]	Clifford Astbury C, Singh J, Naro SG, Boily-Larouche G, Poirier MJP, Emdin F, et al. Strengthening governance for antimicrobial resistance: a One Health perspective. *Rev. Sci. Tech.*;2025;44. doi: 10.20506/rst.44.3675.

[8]	Evidence-informed Policy Network (EVIPNet). Available from: https://www.who.int/initiatives/evidence-informed-policy-network. Accessed 2026 Apr. 27.

[9]	Everybody’s Business: Strengthening Health Systems to Improve Health Outcomes. Geneva; 2007. Available from: https://iris.who.int/server/api/core/bitstreams/809f813f-5b90-4187-861b-3953bb54e244/content. Accessed 2026 Apr. 27.

[10]	The Paris Declaration on Aid Effectiveness and the Accra Agenda for Action. Paris: Organisation for Economic Co-operation and Development; 2005. Available from: https://www.effectivecooperation.org/sites/default/files/documents/Paris%20Declaration%20on%20Aid%20Effectiveness%20.pdf. Accessed 2026 Apr. 27.

[11]	A guide to the World Bank 3rd Edition. Washington, D.C.: World Bank; 2011. Available from: https://documents1.worldbank.org/curated/en/406871468139488518/pdf/638430PUB0Exto00Box0361527B0PUBLIC0.pdf. Accessed 2026 Apr. 27.
